# Dietary Intake of Flavonoids and Carotenoids Is Associated with Anti-Depressive Symptoms: Epidemiological Study and In Silico—Mechanism Analysis

**DOI:** 10.3390/antiox11010053

**Published:** 2021-12-27

**Authors:** Seon-Joo Park, Varun Jaiswal, Hae-Jeung Lee

**Affiliations:** 1 Department of Food and Nutrition, College of BioNano Technology, Gachon University, Gyeonggi 13120, Korea; chris0825@gachon.ac.kr (S.-J.P.); varunjais1@gachon.ac.kr (V.J.); 2 Institute for Aging and Clinical Nutrition Research, Gachon University, Gyeonggi 13120, Korea; 3 Department of Health Sciences and Technology, GAIHST, Gachon University, Incheon 21999, Korea

**Keywords:** flavonoids, carotenoids, depression, in silico

## Abstract

Flavonoids and carotenoids are bioactive compounds that have protective effects against depressive symptoms. Flavonoids and carotenoids are the two main types of antioxidant phytochemicals. This study investigated the association between flavonoid and carotenoid intake and depressive symptoms in middle-aged Korean females. We analyzed the mechanism of these associations using an in silico method. Depressive symptoms were screened using the Beck Depression Inventory-II (BDI-II), and flavonoid and carotenoid intake were assessed using a semi-quantitative food frequency questionnaire. Using a multivariate logistic regression model, we found that flavones, anthocyanins, individual phenolic compounds, lycopene, and zeaxanthin were negatively associated with depressive symptoms. In silico analysis showed that most flavonoids have high docking scores for monoamine oxidase A (MAOA) and monoamine oxidase B (MAOB), which are two important drug targets in depression. The results of the docking of brain-derived neurotrophic factor (BDNF) and carotenoids suggested the possibility of allosteric activation of BDNF by carotenoids. These results suggest that dietary flavonoids and carotenoids can be utilized in the treatment of depressive symptoms.

## 1. Introduction

Depression is one of the most common mental illnesses that affect a person’s poor performance in education, work, and family life [1]. Several studies have reported that depressive symptoms are associated with an elevated risk of cardiovascular disease and heart failure [2,3]. In addition, the risk of depression is elevated in diseases such as angina, arthritis, asthma, cancer, and diabetes [4,5]. In a systematic analysis, depression was found to be associated with various chronic diseases, and the comorbidity of depression deteriorates health compared to depression alone [5]. The depression and depressive symptoms were also found to be associated with all-cause mortality and especially with cardiovascular diseases (CVD) mortality in studies conducted on the different races and populations [6,7]. There is a high prevalence of major depressive disorders, with approximately one in every 20 individuals affected. Up to 85% of residents in low- and middle-income countries did not receive treatment for mental disorders [8]. Therefore, effective prevention and treatment strategies are required to overcome depression. Several studies have shown that the antidepressant effects of polyphenols, especially dietary polyphenols, have the potential to be widely used in depression worldwide because their antidepressant effects can be cost-effective [9,10,11,12]. Most dietary polyphenols are associated with reduced symptoms of depression, and consumption of some polyphenols significantly reduces depressive symptoms [9]. Flavonoids are an important class of polyphenols with antidepressant properties reported in several research and review papers [13,14,15,16]. The anti-depressive effect of dietary carotenoids has also been observed in several studies [17,18]. Similarly, low concentrations of carotenoids in the blood are associated with depressive symptoms [19]. Therefore, the identification of dietary flavonoids and carotenoids with anti-depression properties and knowledge of the possible mechanisms are of great importance in establishing the use of flavonoids and carotenoids for depression prevention. Depression is a complex disorder, and different mechanisms are thought to explain its pathophysiology. These mechanisms include the biogenic amine (monoamine) hypothesis, dysregulation of the hypothalamic–pituitary–adrenal axis, abnormalities in the function of receptors (such as 5-hydroxytryptamine 1 (5-HT1), 5-HT2, and alpha2-adrenoceptors), neuroinflammation [20], genetic factors [21] antioxidant effects and anti-neuro-inflammation [16], and immune and environmental factors [22]. Other possible mechanisms for depression may include a lack or decrease in adult neurogenesis [23], abnormalities in the second messenger system, and elevated levels of corticotrophin-releasing factor (CRF) [24]. However, the exact mechanism underlying the initiation and progression of depression is unknown. Various natural flavonoids and carotenoids have been studied in various in vivo studies for their antidepressant properties in rats and mice, with mostly positive outcomes [25]. Natural flavonoids derived from plants such as rutin [26], quercetin [27,28], apigenin [29], epigallocatechin gallate [30], myricetin [31], hesperidin [32], kaempferol [28], naringenin [33], formononetin [34], beta-carotene [35], beta-cryptoxanthin [36], lutein [37] and genistein [38] have been shown to function as antidepressants in animal models. Recently, a high intake of dietary flavonoids has been associated with decreased depressive symptoms and improved general mental health in human studies [11,13].

To date, limited studies have been conducted to explore the possible mechanisms of the association of individual dietary flavonoid and carotenoid intake with antidepressant outcomes using an in silico analysis. Therefore, this study aimed to evaluate the effects of dietary flavonoid and carotenoid intake on depressive symptoms in middle-aged Korean females. In addition, flavonoids and carotenoids have also been used to explore possible mechanisms of action in molecular docking studies using an in silico analysis.

## 2. Materials and Methods

### 2.1. Subjects

The population in this diet-depression cohort study comprised participants recruited through hospital and community health centers in the Seoul and Gyeonggi areas of South Korea. This study was conducted from 2016 to 2018. Finally, 2201 females aged 45–69 years participated in the baseline survey. We estimated the sample size using STATCALS (https://www.cdc.gov/epiinfo/user-guide/statcalc/cohortandcrosssectional.html accessed on 10 April 2016) and previously study [39] (α = 0.05, β = 0.2, OR=0.65, prevalence 15%, drop rate 25%). Informed consent was obtained from all participants involved in the study. We excluded participants with implausible energy intakes [40] of <500 kcal/day (*n* = 8) and >3500 kcal/day (*n* = 3). As a result, a total of 2190 data were used for the final analysis. 

The study was approved by the Institutional Review Board of the Gachon University Gil Medical Center (GDIRB2016-271) and was conducted in accordance with the Declaration of Helsinki.

### 2.2. Methods

#### 2.2.1. Depressive Disorder Screening

Depressive disorder screening was conducted using the Beck Depression Inventory-II (BDI-II) and Center for Epidemiologic Studies-Depression Scale (CES-D). The BDI-II contains 21 questions, with each answer scored on a scale of zero to three, for a total score between zero and 63. A higher score was associated with severe depressive symptoms [41]. The Korean version of the BDI-II validated tool was used to assess depressive symptoms. We classified people with a BDI-II score of 14 or higher as those with depressive symptoms. The CES-D questionnaire consists of 20 questions, with a total score ranging from zero to 60, with higher scores indicating more severe depressive symptoms. Subjects with a CES-D score of 16 or higher were considered to have depressive symptoms [42]

#### 2.2.2. Nutritional Assessment

Dietary intake, including macro-nutrient intake and flavonoid/carotenoid intake per day, were assessed using the previously validated 108-item semi-quantitative food frequency questionnaire (SQ-FFQ) [43]. The frequency of food intake was assessed over nine categories (three times/day, two times/day, one time/day, five to six times/week, two to four times/week, one time/week, two to three times/month, one time/month, almost never), and serving size was assessed as 0.5, 1, or 1.5 serving. Nutrient intake was calculated using the food composition database created by the Rural Development Administration of Korea [44]. Flavonoid and carotenoid content in foods was obtained from the tables of food functional composition by the National Academy of Agricultural Sciences of Korea [45] and the United States Department of Agriculture (USDA) database [46,47,48].

The subclass and individual phenolic compound of flavonoids are as follows: flavonols (kaempferol, myricetin, quercetin), flavones (luteolin, apigenin), flavanols, ((+)-catechin, (+)-gallocatechin, (−)-epicatechin, (−)-epigallocatechin, (−)-epicatechin 3-gallate, theaflavin, theaflavin 3-gallate, theaflavin 3′-gallate, theaflavin 3,3′ digallate), flavanones (hesperptin, naringenin, eriodictyol), isoflavones (daidzein, genistein, glycitein, coumestrol, formonnetin, biochanin A), anthocyanins (cyanidin, delphinidine, pelargonidine, peonidine). We defined total flavonoids as the sum of all these subclasses. In addition, the intake of flavonols, flavones, flavanols, flavanones, isoflavones, and anthocyanins were summed up for individual phenolic compound intakes. Carotenoids and subclasses of carotenoids are as follows: α-carotene, β-carotene, lycopene, lutein, zeaxanthin, β-cryptoxanthin, and capsaicin. We defined total carotenoids as the sum of these subclasses.

#### 2.2.3. Other Variables

The subject’s height and weight were measured to the nearest 0.1 cm and 0.1 kg, respectively. The body mass index (BMI) was calculated as the weight in kilograms/height in meters squared.

The general characteristics and lifestyle data of the subjects were collected through face-to-face interviews via questionnaires. We considered education level (elementary school graduation or less, middle school graduation, high school graduation, and college graduation or higher), household income (<1000 dollar, 1000–2000 dollar, 2000–4000 dollar, >4000 dollar), current smoking (yes or no), current alcohol drinking (yes or no), marital status (married or other). The job type (white-collar worker, service worker, blue-collar worker, or housewife), chronic disease such as diabetes, hypertension, heart disease, or cancer (yes or no), physical activity (yes or no), menopausal status (yes or no), family history of depression (yes or no), use of antidepressant (yes or no), sleep duration (<6 h, 6–8 h, >8 h), stress (rarely, a litter, a lot, very much), age, BMI, and energy intake as potential confounding factors.

#### 2.2.4. Molecular Docking (In Silico Analysis)

The crystal structures of three important targets in depression, monoamine oxidase A (MAOA) PDBID: 2Z5X [49], monoamine oxidase B (MAOB) PDBID: 4A79 [50] and brain-derived neurotrophic factor (BDNF) PDBID: 1B8M), were obtained from the PDB database. Inhibitors and molecules other than proteins were removed from the MAOA and MAOB structures, but flavin adenine dinucleotide (FAD), the co-enzyme present in MAOA and MAOB structures, was retained in the active site to mimic natural conditions. Proteins were prepared, and the docking grid was defined around the active site, which was the binding site of the known inhibitor (harmine and pioglitazone were the inhibitors present in MAOA and MAOB crystal structures, respectively [49,50]) using the AutoDock (version 4.2) Tool Kit (ADT version 1.5.6) and AutoGrid (version 4) [6], respectively. In the case of BDNF, the binding cavity was predicted using the CavityPlus web server [51]. The docking grid was defined to surround all predicted binding site residues that were distant from the loop region using AutoGrid through ADT. The compounds used in the docking study as ligands were extracted from PubChem, and format conversion was carried out using babel for the docking study [52]. Further ligand preparation was performed using ADT [53]. In the docking simulation, the number of evaluation steps was increased to 25,000,000 because some compounds had ten or more rotatable bonds, and finally, docking was carried using AutoDock 4.2 [53]. The binding affinity of ligands with targeted proteins was determined by the estimated free binding energy of binding (EFEB), which is considered to be better with a higher negative value. Interaction studies of ligands in protein–ligand complexes were carried out using LigPlot plus [54] and Chimera [55].

#### 2.2.5. Statistical Analysis

The characteristics of the study subjects were expressed as the mean and standard deviation for continuous variables or as percentages for categorical variables. The differences between the control and depressive symptom groups were analyzed using independent *t*-tests (continuous variables), Mann–Whitney test (intakes of flavonoids and carotenoids), and chi-squared tests (categorical variables). The association between flavonoid and carotenoid intake and depressive symptoms using multivariable logistic regression analysis, education level, household income, marital status, age, BMI, job status, drinking, smoking, physical activity, family history of depression, stress, chronic disease status, sleep duration, menopause, and total energy intake were considered confounding factors. Flavonoid and carotenoid intake were categorized into quartiles, with the lowest quartile group considered as the reference group. Pearson correlation coefficient between the docking score of MAOA and MAOB was calculated using the SAS.

All statistical analyses were performed using SAS software (version 9.4 SAS institute Inc., Cary, NC, USA), and statistical significance was set at a *p*-value of <0.05.

## 3. Results

### 3.1. Characteristics of the Study Participants

The characteristics of the participants are summarized in Table 1. The sample comprised 2190 individuals with a mean age of 58.2 years (range 45–69 years), and the mean BMI was 24.1. Among the 2190 participants, 487 individuals (22.2%) were identified as having depressive symptoms using the BDI-II questionnaire (BDI-II score ≥ 14, mean score: 21.0 ± 7.0), and 363 subjects (16.6%) were classified as having depressive symptoms using the CES-D questionnaire (CES-D score ≥ 16, mean score: 23.7 ± 7.7).

### 3.2. Flavonoid and Carotenoid Intake between the Control and Depressive Symptoms Groups

Table 2 shows the intake of phytochemicals between control and depressive symptoms groups. The depressive symptoms group was classified as those with a BDI-II score of 14 or higher. The intake of total flavonoids was lower in the depressive symptom group, and similar results were observed in the flavonols, flavones, flavanols, flavanones, isoflavones, and anthocyanins. Subgroups of these compounds, the intake of myricetin, quercetin, luteolin, eriodictyol, daidzein, genestein, glycitein, coumesterol, cyanidin, delphinidine, pelargonidine, peonidin were lower in the depressive symptoms group than in the control group. The intakes of total carotenoids and most carotenoid subclasses (α-carotene, β-carotene, lycopene, lutein, zeaxanthin, β-cryptoxanthin, capsaicin) were significantly lower in the depressive symptoms group than in the control group.

### 3.3. Association between Flavonoid Intake and Depressive Symptoms

Table 3 presents the results of the multivariable-adjusted regression analysis, showing that the risk of depressive symptoms was negatively associated with flavonoid intake, especially flavones (OR = 0.69, 95% CI: 0.48–0.99, *p* for trend = 0.0388) and anthocyanins (OR = 0.68, 95% CI: 0.48–0.96, *p* for trend = 0.0093) after adjusting for confounding factors. However, the total flavonoid intake was not statistically significant (OR = 0.69, 95% CI: 0.47–1.02, *p* for trend = 0.0604).

Adjusted ORs and 95% CIs of depressive symptoms related to individual phenolic compound intake are summarized in Table 4. In model 1, myricetin, luteolin, (+)-catechin, (+)-gallocatechin, (−)-epicatechin, (−)-epigallocatechin, theaflavin, theaflavin 3-gallate, theaflavin 3′-gallate, theaflavin 3,3′ -digallate, naringenin, eriodictyol, daidzein, genistein, cyanidin, pelargonidine, and peonidine showed significant linear relationships. In model 2, compared with subjects in the lowest quartile of the phenolic compound intake, those in the highest quartile had a significantly lower odds of depressive symptoms (OR = 0.57, 95% CI: 0.39–0.82, *p* for trend = 0.004 for luteolin; OR = 0.65, 95% CI: 0.45–0.94, *p* for trend = 0.0128 for (+)-catechin; OR = 0.73, 95% CI: 0.53–1.00, *p* for trend = 0.0226 for (+)-gallocatechin; OR = 0.69, 95% CI: 0.48–0.98, *p* for trend = 0.0395 for theaflavin 3-gallate, OR = 0.66, 95% CI: 0.47–0.93, *p* for trend = 0.0055 for cyanidin; OR = 0.64, 95% CI: 0.46–0.90, *p* for trend = 0.0199 for pelargonidine).

### 3.4. Association between Carotenoid Intake and Depressive Symptoms

Table 5 shows that the association between depressive symptoms and total carotenoids and subclass of carotenoid intake. Lycopene and zeaxanthin were associated with lower prevalence of depressive symptoms after adjusting for multiple confounding factors (OR = 0.66, 95% CI: 0.47–0.92, *p* for trend = 0.0106 for lycopene and OR = 0.63, 95% CI: 0.44–0.90, *p* for trend = 0.028 for zeaxanthin, respectively).

### 3.5. Results of the In Silico Analysis

**Figure 1:**
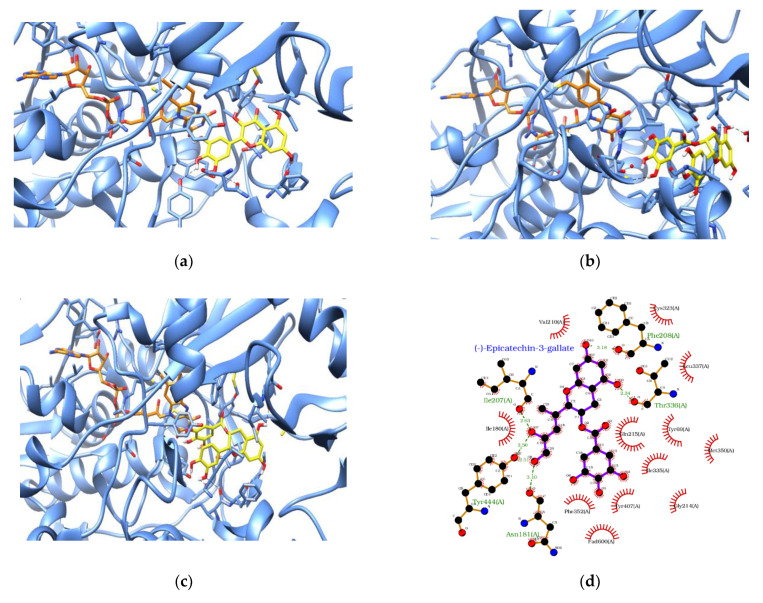

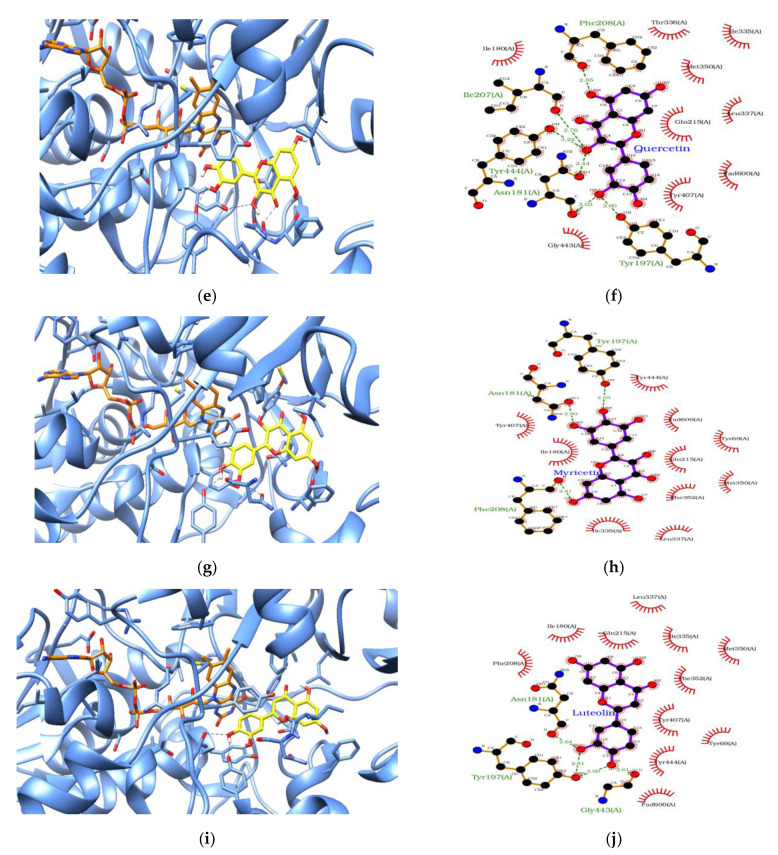
(**a**): MAOA active site with ligand and co-enzyme (protein MAOA is shown in blue, compound in yellow and FAD co-enzyme in orange). (**b**) MAOB active site with ligand and co-enzyme (protein MAOB is shown in blue, compound in yellow and FAD co-enzyme in orange). (**c**,**d**) Interaction between the first ranked compound ((−)-epicatechin-3-gallate) with the target protein. (**e**,**f**) Interaction between the second-ranked compound (quercetin) with the target protein. (**g**,**h**) Interaction between the third-ranked compound (myricetin) with the target protein. (**i**,**j**) Interaction between the fourth-ranked compound (luteolin) with the target protein. (In 3D interaction figures, MAOA and MAOB are shown in blue, compounds in yellow, and FAD co-enzyme in orange).

Docking results of all selected flavonoid and carotenoid compounds with MAO enzymes (MAOA and MAOB) and BDNF, respectively, were ranked according to the docking score, that is, EFEB. The top-scoring ligand in MAOA and MAOB was (−)-epicatechin-3-gallate, which had docking scores of −12.73 and −13.84, respectively (Table 6). A positive correlation between the EFEB docking scores of MAOA and MAOB 0.509 was observed. In the case of BDNF, the top-scoring molecule was alpha-carotene with an EFEB value of −7.24, and the minimum EFEB was −6.06 for lutein (Table 7). Further interaction studies of the top-scoring molecules in docked ligand–protein complexes of MAOA and MAOB revealed a similar binding pose. Multiple hydrogen bonds and hydrophobic interactions were present between the protein and ligand complexes (Figure 1). According to EFEB in MAOA, the top four flavonoids were (−)-epicatechin-3-gallate, quercetin, myricetin, and luteolin, which had 6 hydrogen bonds (HB) and 12 hydrophobic interactions (HPhoI), 4 HB and 9 HPhoI, 3 HB and 10 HPhoI, and 4 HB and 11 HPhoI, respectively (Figure 1 and Table 6).

**Figure 2:**
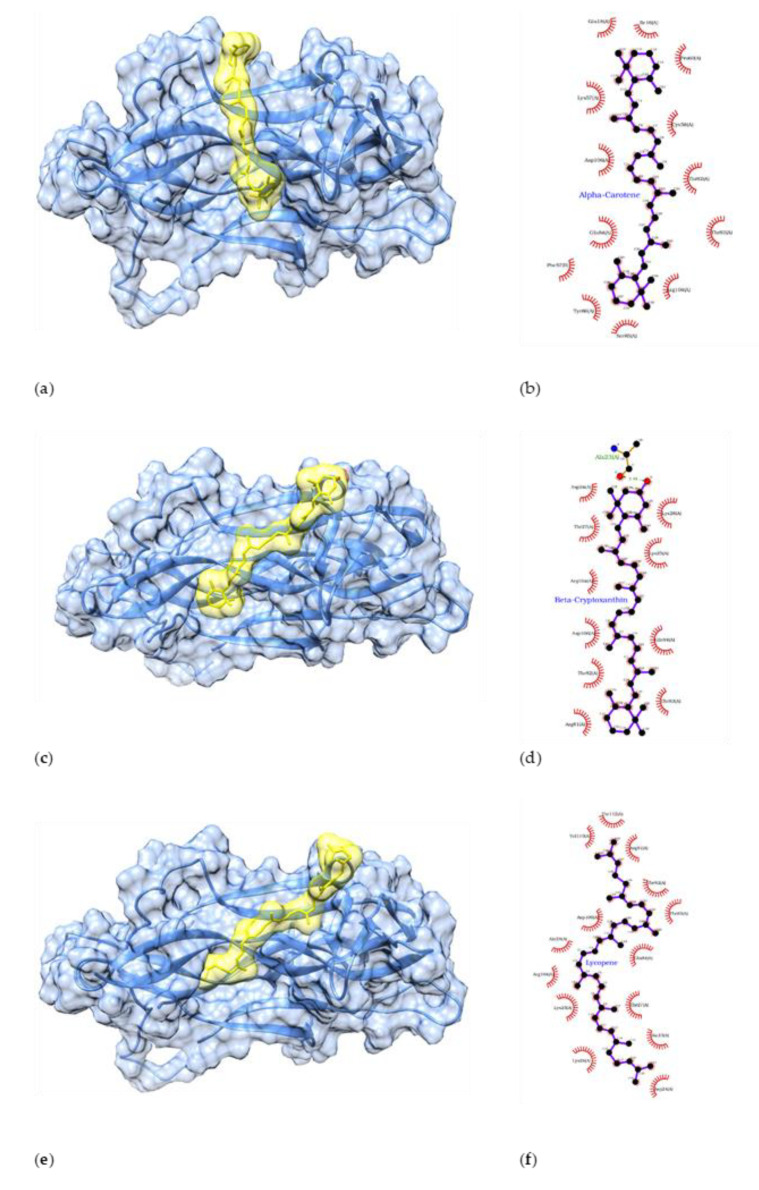
Interaction between the first ranked compound (α-carotene) with BDNF (**a**,**b**). Interaction between the second-ranked compound (β-cryptoxanthin) with the target BDNF protein (**c**,**d**). Interaction between the third-ranked compound (lycopene) with BDNF protein (**e**,**f**). In 3D interaction figures, BDNF is shown in blue and compounds in yellow).

Similarly, an interaction study of BDNF with docked ligands revealed multiple hydrophobic interactions in protein–ligand complexes. A total of 13, 10, and 14 hydrophobic interactions were found for α-carotene, β-cryptoxanthin, and lycopene in the protein–ligand docked complex, and five residues (Thr82, Thr83, Gln84, Arg104, and Asp106) were found to be common among these three top-scoring ligands (Figure 2). As per the defined binding cavity, the ligands were docked in the middle region of the protein (Figure 2).

## 4. Discussion

This study was conducted to investigate the association between the dietary intake of flavonoids and carotenoids and depressive symptoms among middle-aged Korean females and to clarify the relevant mechanisms of the association using in silico analysis. To date, a variety of dietary flavonoids and carotenoids have shown antidepressant properties in numerous studies [13,15,16,26,29]. Additionally, these flavonoids and carotenoids are abundant in food and have potential therapeutic activities for depression, and can be used as a cost-effective means [14].

Although the antidepressant properties of several flavonoids and carotenoids have been studied to date, the complex nature of depression, including different mechanisms and pathways, hinders an accurate understanding of the mechanisms of antidepressant action [10,56]. However, different results of the in vivo studies suggest that some major flavonoids/carotenoids must be explored for their anti-depression effects and the most probable mechanisms for the development of antidepressants in the population [7,9,11].

In this study, subjects with depressive symptoms had fewer intakes of total flavonoids, subclass flavonoids, and individual flavonoids than control subjects. An intervention study reported that a high polyphenol diet (including six portions of fruit and vegetables and 50 grams of dark chocolate/day) for eight weeks reduced depressive symptoms in patients with mild hypertension [11]. In addition, an inverse association between subclass flavonoid (flavonol, flavone, and flavoanone) intake and depression risk has also been reported in a cohort study of middle-aged and older females in the United States [13]. A cross-sectional study showed that the highest dietary phytochemical index group had a lower prevalence of depressive symptoms among females in Iran [57].

In a multivariate-adjusted logistic regression analysis, dietary intake of flavones and anthocyanin subclasses was negatively associated with the risk of depressive symptoms in this study. Similarly, a recent study reported that flavanols, flavonols, flavononoes, flavones, and anthocyanin subclasses were inversely associated with depressive symptoms in adults living in the Mediterranean region [9]. However, the isoflavone subclass did not show any association with depressive symptoms, as observed in our study.

Among the individual compounds of flavonoids, luteolin, (+)-catechin, (+)-gallocatechin, theaflavin, and theaflavin 3-gallatecyanidin and pelargonidine were negatively associated with the risk of depressive symptoms in a logistic regression analysis. Naringin and quercetin intake were negatively associated with depression in a Mediterranean study [9].

Among the subclasses of carotenoids, lycopene and zeaxanthin intake showed significantly (34% and 37%, respectively) lower depressive symptom risk in this study. In the United States National Health and Nutrition Examination Survey, total carotenoid and all subgroup carotenoid intakes were inversely associated with depressive symptoms [17]. A cross-sectional study reported that α-carotene and β-carotene intake were inversely associated with the CES-D score [18]. In animal studies, lycopene administration (60 mg/kg) decreased plasma levels of lipopolysaccharide (LPS)-induced interleukin-1β (IL-1β) and heme oxygenase-1 (HO-1) and decreased interleukin-6 (IL-6) and tumor necrosis factor-α (TNF-α) in plasma [58]. In another animal study, IL-6, interleukin-1β (IL-1β), and TNF-α in the hippocampus were reduced by zeaxanthin treatment [59]. These results suggest that carotenoids could be used as potential therapeutics.

Individual compounds from flavonoids and carotenoids have been tested for mechanisms in important drug targets, MAO (MAOA and MAOB) and BDNF [15,50,60,61,62,63], which are known to be associated with depression through molecular docking studies for possible inhibitory roles. In flavonoids, MAO inhibition was selected to study the mechanism of anti-depression as some flavonoids are known to inhibit the MAO enzyme in the literature [57,59,61]. However, the docking score of the selected flavonoids had positive correlations in MAOA and MAOB, which is in line with the literature suggesting that a similar binding pocket is present in both these targets [64]. However, most of the individual flavonoid compounds showed high negative values of EFEB in MAOA and MAOB docking, which could be due to their inhibitory role in these enzymes. Contrary to the epidemiological data analysis results, theaflavin and theaflavin-3-gallate achieved high EFEB in the molecular docking study, suggesting their inability to inhibit MAOA and MAOB as drug targets. The high molecular weight (>500) of these flavonoids is expected to be the reason for the inability of these compounds to interact optimally with MAO. Therefore, these flavonoids may have different modes of action for their antidepressant properties, as intake of theaflavin and theaflavin 3-gallate are negatively associated with the risk of depressive symptoms (Table 4). Flavonoids with high negative values of EFEB have a high possibility of inhibiting MAO and the mode of anti-depression action through the MAO enzyme. Compounds with a docking score better than -9 EFEB could inhibit MAO as one of the important mechanisms for anti-depressive effects. Similar docking poses of molecules inside the binding pocket (Figure 1) and the literature also support our results, as flavonoids such as quercetin, luteolin, biochanin A, and cyanidine are known to inhibit MAO [58,59,62]. The top-scoring molecule, that is, (−)-epicatechin-3-gallate, has bioavailability issues as it has low absorption in the stomach when taken with food [65]. Bioavailability could be the main reason for the lack of significant association of (−)-epicatechin-3-gallate with anti-depressive symptoms in our study [65]. (−)-epicatechin-3-gallate is mainly contained in green tea. In our study, the intake of green tea, citron tea, and black tea was combined, so the intake of green tea may be underestimated. Furthermore, other compounds that had better docking scores but were not found to be significantly associated with depressive symptoms in our statistical analysis could be due to limitations of this study such as the small number of subjects and cross-sectional study design. Different flavonoids were found to be associated with direct and indirect mechanisms in the pathophysiology of depression, which is an active area of research for the development of therapeutics [66]. A complex mechanistic point was explored here to study the possible inhibition of MAOA and MAOB, which are known targets of flavonoids for anti-depressive effects in several cases [15,50,60,61,62]. The bioavailability of flavonoids is another important point because limited information regarding the bioavailability (reach of different flavonoids in the central nervous system) potential is not known [67]. Nevertheless, bioavailability and other important factors such as absorption, metabolism, and distribution of flavonoids are important questions to be considered in future research.

In the case of carotenoids, all ligands were docked in the middle part of BDNF, which is distant from the N-terminal region involved in tropomyosin receptor kinase B (TrkB) binding [68]. Hence, the binding of carotenoids does not directly interfere with TrkB binding and is expected to exert an allosteric effect on the TrkB binding region. However, in vitro experiments are required to confirm allosteric activation of the system. The top-scoring compound was α-carotene in the BDNF and carotenoid docking studies. However, all six carotenoids had slight differences in their docking scores (Table 7), which suggests a similar binding affinity of these carotenoids to BDNF.

The current study is meaningful in that it is the first to analyze the association between the intake of flavonoids and carotenoids and depressive symptoms using epidemiologic data and investigate the mechanism using an in silico analysis. This study also had several limitations. Because of its cross-sectional design, a causal relationship between the intake of flavonoids/carotenoids and depressive symptoms has not been identified. The docking results suggested that compounds with molecular weights greater than 500 could not inhibit MAO enzymes [15,50,60,61,62]. These results suggest that dietary flavonoids and carotenoids can be utilized in the treatment of depressive symptoms.

## Figures and Tables

**Table 1:** Characteristics of the subjects.

Variables	Total (*n* = 2190)
Age (years), mean ± SD	58.2 ± 5.8
BMI (kg/m^2^), mean ± SD	24.1 ± 3.2
BDI-II score, mean ± SD	9.0 ± 7.8
CES-D score, mean ± SD	8.5 ± 8.5
Depressive disorder prevalence, *n* (%)	
BDI-II score ≥ 14	487 (22.2)
CES-D score ≥ 16	363 (16.6)
Stage of depression using BDI-II score, *n* (%)	
Minimal (0–13)	1703 (77.8)
Mild (14–19)	270 (12.3)
Moderate (20–28)	151 (6.9)
Severe (29–63)	66 (3.0)
Stage of depression using CES-D score, *n* (%)	
Normal (0–15)	1827 (83.4)
Probable depression (16–24)	238 (10.9)
Definite depression(25–60)	125 (5.7)
Education level, *n* (%)	
Elementary school	322 (14.7)
Middle school	569 (25.9)
High school	967 (44.2)
College and higher	332 (15.2)
Household income, *n* (%)	
<1000 dollar	184 (8.4)
1000–2000 dollar	450 (20.5)
2000–4000 dollar	801 (36.6)
>4000 dollar	755 (34.5)
Current Smoking, *n* (%)	
No	2119 (96.8)
Yes	71 (3.2)
Current alcohol drinking, *n* (%)	
No	1530 (69.9)
Yes	660 (30.1)
Physical activity, *n* (%)	
No	907 (41.4)
Yes	1283 (58.6)
Marital status, *n* (%)	
Married	1689 (77.1)
Others	501 (22.9)
Job, *n* (%)	
White-collar worker	169 (7.7)
Service worker	497 (22.7)
Blue-collar worker	205 (9.4)
Housewife	1319 (60.2)
Chronic disease, *n* (%)	
No	1440 (65.8)
Yes	750 (34.2)
Family history of depression, *n* (%)	
No	2150 (98.2)
Yes	40 (1.8)
Use of antidepressant, *n* (%)	
No	2154 (98.4)
Yes	36 (1.6)
Sleep duration, *n* (%)	
<6 h	385 (17.5)
6–8 h	1420 (65.0)
>8 h	385 (17.5)
Stress, *n* (%)	
Rarely	549 (25.1)
A litter	1088 (49.7)
A lot	520 (23.7)
Very much	33 (1.5)
Menopausal status	
No	282 (12.9)
Yes	1908 (87.1)

SD, standard deviation; BDI-II, Beck Depression Inventory-II; CES-D, Center for Epidemiological Studies-Depression Scale. Chronic disease: diabetes, hypertension, heart disease, or cancer diagnosis.

**Table 2:** Intake of flavonoids and carotenoids between the control and depressive symptom groups.

Variables	Control (*n* = 1703)	Depressive Symptoms * (*n* = 487)	*p*-Value
Total flavonoids	126.12 ± 1.44	113.71 ± 2.71	<0.0001
Flavonols	13.39 ± 0.19	12.84 ± 0.38	0.0174
Flavones	1.45 ± 0.02	1.30 ± 0.03	<0.0001
Flavanols	78.90 ± 1.08	70.51 ± 2.05	0.0001
Flavanones	7.44 ± 0.19	6.82 ± 0.31	0.046
Isoflavonoids	15.87 ± 0.30	14.68 ± 0.52	0.0322
Anthocyanins	8.74 ± 0.19	7.29 ± 0.31	0.0001
Flavonols			
Kaempferol	1.53 ± 0.0.02	1.49 ± 0.05	0.2623
Myricetin	0.18 ± 0.00	0.17 ± 0.00	0.0004
Quercetin	11.69 ± 0.17	11.18 ± 0.34	0.0149
Flavones			
Luteolin	1.23 ± 0.01	1.09 ± 0.03	<0.0001
Apigenin	0.22 ± 0.00	0.21 ± 0.01	0.093
Flavanols			
(+)-Catechin	3.71 ± 0.06	3.22 ± 0.11	<0.0001
(+)-Gallocatechin	0.01 ± 0.00	0.01 ± 0.00	0.003
(−)-Epicatechin	3.37 ± 0.07	2.88 ± 0.13	<0.0001
(−)-Epigallocatechin	0.30 ± 0.01	0.27 ± 0.01	0.0003
(−)-Epicatechin 3-gallate	0.01 ± 0.00	0.01 ± 0.00	0.8674
Theaflavin	9.67 ± 0.16	8.41 ± 0.29	<0.0001
Theaflavin 3-gallate	6.21 ± 0.09	5.50 ± 0.17	<0.0001
Theaflavin 3′-gallate	26.13 ± 0.36	23.43 ± 0.70	0.0003
Theaflavin 3,3′ digallate	29.49 ± 0.40	26.79 ± 0.78	0.0016
Flavanones			
Hesperidin	6.58 ± 0.17	6.11 ± 0.30	0.0515
Naringenin	0.83 ± 0.03	0.69 ± 0.03	0.0012
Eriodictyol	0.03 ± 0.00	0.03 ± 0.00	0.0215
Isoflavones			
Daidzein	6.07 ± 0.12	5.63 ± 0.21	0.0487
Genistein	7.58 ± 0.14	6.99 ± 0.25	0.0251
Glycitein	2.12 ± 0.04	1.98 ± 0.07	0.0436
Coumestrol	0.06 ± 0.00	0.06 ± 0.00	0.0216
Formonnetin	0.01 ± 0.00	0.01 ± 0.00	0.1729
Biochanin A	0.01 ± 0.00	0.01 ± 0.00	0.1777
Anthocyanins			
Cyanidin	7.04 ± 0.17	5.80 ± 0.26	<0.0001
Delphinidine	0.32 ± 0.02	0.28 ± 0.03	0.0019
Pelargonidine	0.21 ± 0.01	0.18 ± 0.01	<0.0001
Peonidine	1.18 ± 0.03	1.04 ± 0.06	0.0046
Total carotenoids	24.69 ± 0.32	22.62 ± 0.59	0.0001
α-carotene	0.53 ± 0.01	0.45 ± 0.02	0.0042
β-carotene	6.46 ± 0.08	5.95 ± 0.15	0.0004
Lycopene	2.21 ± 0.06	1.91 ± 0.10	0.0015
Lutein	1.90 ± 0.03	1.79 ± 0.06	0.0391
Zeaxanthin	0.21 ± 0.00	0.19 ± 0.01	0.0038
β-cryptoxanthin	0.31 ± 0.00	0.28 ± 0.01	0.0025
Capsaicin	12.40 ± 0.20	11.44 ± 0.38	0.0015

* Depressive symptoms: BDI-II score ≥ 14; Values expressed as mean ± SE (standard error).

**Table 3:** Association between total flavonoids and subclass intake and prevalence of depressive symptoms in multivariate-adjusted logistic regression analysis.

Variables	Quartile	Median	No. of Total	No. of Cases	Model 1			Model 2		
					OR	95% CI Lower Upper		OR	95% CI Lower Upper	
Total flavonoids	Q1	58.1	547	147	1.00			1.00		
	Q2	99.7	548	133	0.88	0.67	1.16	0.99	0.72	1.35
	Q3	135.9	548	111	0.70	0.53	0.93	0.92	0.65	1.29
	Q4	193.7	547	96	0.59	0.44	0.79	0.69	0.47	1.02
*p*-value for trend					0.0001			0.0604		
Flavonols	Q1	6.2	547	139	1.00			1.00		
	Q2	10.1	548	120	0.83	0.63	1.10	0.95	0.69	1.31
	Q3	13.8	548	110	0.75	0.56	1.00	0.99	0.70	1.39
	Q4	20.4	547	118	0.81	0.61	1.08	1.05	0.73	1.53
*p*-value for trend					0.1602			0.7063		
Flavones	Q1	0.7	547	158	1.00			1.00		
	Q2	1.2	548	124	0.73	0.55	0.95	0.86	0.63	1.17
	Q3	1.6	548	106	0.60	0.45	0.79	0.78	0.57	1.09
	Q4	2.2	547	99	0.55	0.41	0.74	0.69	0.48	0.99
*p*-value for trend					<0.0001			0.0388		
Flavanols	Q1	21.7	547	148	1.00			1.00		
	Q2	61.0	548	121	0.77	0.58	1.02	0.96	0.70	1.31
	Q3	87.9	548	124	0.80	0.61	1.06	1.12	0.81	1.55
	Q4	128.5	547	94	0.57	0.42	0.77	0.77	0.54	1.11
*p*-value for trend					0.0004			0.2801		
Flavanones	Q1	1.2	547	138	1.00			1.00		
	Q2	3.4	548	112	0.76	0.58	1.01	0.82	0.60	1.13
	Q3	7.7	548	126	0.89	0.67	1.17	0.83	0.60	1.13
	Q4	13.5	547	111	0.76	0.57	1.00	0.80	0.57	1.12
*p*-value for trend					0.1707			0.2967		
Isoflavones	Q1	4.9	547	137	1.00			1.00		
	Q2	9.6	548	128	0.91	0.69	1.21	0.94	0.68	1.28
	Q3	15.7	548	115	0.79	0.59	1.05	0.75	0.54	1.04
	Q4	28.8	547	107	0.73	0.55	0.97	0.73	0.51	1.07
*p*-value for trend					0.0217			0.0784		
Anthocyanins	Q1	1.9	547	141	1.00			1.00		
	Q2	4.4	548	135	0.95	0.72	1.25	1.02	0.75	1.40
	Q3	9.0	548	114	0.76	0.58	1.01	0.83	0.60	1.16
	Q4	16.6	547	97	0.63	0.47	0.84	0.68	0.48	0.96
*p*-value for trend					0.0006			0.0093		

Model 1 adjusted for age; model 2 adjusted for age, BMI, education level, household income, marital status, job, current alcohol drinking, current smoking, physical activity, chronic disease status (diabetes, hypertension, cancers, or cardiovascular diseases), sleep duration, family history of depression, stress, menopause status, and total energy intake; OR, odds ratio; CI, confidence interval; Q, quartile.

**Table 4:** Association between individual phenolic compound intake and prevalence of depressive symptoms in multivariate-adjusted logistic regression analysis.

Variables	Quartile	Median	No. of Total	No. of Cases	Model 1			Model 2		
					OR	95% CI Lower Upper		OR	95% CI Lower Upper	
Flavonols										
Kaempferol	Q1	0.61	547	132	1.00			1.00		
	Q2	1.10	548	115	0.84	0.63	1.12	1.05	0.76	1.44
	Q3	1.59	548	121	0.89	0.67	1.18	1.13	0.82	1.55
	Q4	2.47	547	119	0.87	0.66	1.15	1.21	0.85	1.71
*p*-value for trend					0.4562			0.2663		
Myricetin	Q1	0.09	547	153	1.00					
	Q2	0.14	548	115	0.69	0.52	0.91	0.83	0.60	1.14
	Q3	0.18	548	124	0.75	0.57	0.99	0.91	0.65	1.26
	Q4	0.26	547	95	0.54	0.41	0.73	0.66	0.44	0.98
*p*-value for trend					0.0001			0.0602		
Quercetin	Q1	5.05	547	139	1.00					
	Q2	8.74	548	119	0.82	0.62	1.09	0.93	0.68	1.28
	Q3	12.12	548	110	0.75	0.56	1.00	1.01	0.72	1.41
	Q4	17.96	547	119	0.82	0.62	1.09	1.06	0.73	1.53
*p*-value for trend					0.1858			0.6646		
Flavones										
Luteolin	Q1	0.59	547	161	1.00					
	Q2	0.96	548	122	0.69	0.53	0.91	0.81	0.60	1.11
	Q3	1.32	548	114	0.64	0.48	0.84	0.83	0.60	1.15
	Q4	1.87	547	90	0.48	0.36	0.64	0.57	0.39	0.82
*p*-value for trend					<0.0001			0.004		
Apigenin	Q1	0.00	547	138	1.00					
	Q2	0.19	548	116	0.80	0.60	1.06	0.81	0.58	1.11
	Q3	0.26	548	116	0.81	0.61	1.07	1.06	0.77	1.46
	Q4	0.39	547	117	0.83	0.62	1.10	1.13	0.81	1.59
*p*-value for trend					0.1531			0.5143		
Flavanols										
(+)-Catechin	Q1	1.23	547	152	1.00			1.00		
	Q2	2.45	548	127	0.79	0.60	1.04	0.98	0.72	1.34
	Q3	3.70	548	114	0.69	0.52	0.92	0.84	0.60	1.16
	Q4	6.38	547	94	0.55	0.41	0.73	0.65	0.45	0.94
*p*-value for trend					<0.0001			0.0128		
(+)-Gallocatechin	Q1	0.00	562	140	1.00			1.00		
	Q2	0.00	381	92	0.96	0.71	1.30	1.02	0.73	1.44
	Q3	0.01	540	123	0.90	0.68	1.19	0.90	0.66	1.24
	Q4	0.02	707	132	0.70	0.54	0.92	0.73	0.53	1.00
*p*-value for trend					0.0069			0.0226		
(−)-Epicatechin	Q1	0.60	547	155	1.00			1.00		
	Q2	1.53	548	126	0.76	0.58	1.00	0.85	0.63	1.16
	Q3	3.31	548	98	0.56	0.42	0.74	0.65	0.47	0.91
	Q4	7.09	547	108	0.63	0.48	0.84	0.77	0.54	1.08
*p*-value for trend					0.0033			0.169		
(−)-Epigallocatechin	Q1	0.09	547	147	1.00			1.00		
	Q2	0.18	548	125	0.81	0.61	1.06	0.85	0.62	1.16
	Q3	0.32	548	115	0.73	0.55	0.97	0.93	0.67	1.29
	Q4	0.54	547	100	0.61	0.46	0.82	0.76	0.53	1.09
*p*-value for trend					0.0011			0.2027		
(−)-Epicatechin 3-gallate	Q1	0.00	547	125	1.00			1.00		
	Q2	0.00	548	113	0.87	0.65	1.16	0.90	0.65	1.25
	Q3	0.01	548	112	0.85	0.64	1.14	1.05	0.76	1.46
	Q4	0.01	547	137	1.10	0.83	1.46	1.36	0.97	1.92
*p*-value for trend					0.6703			0.0742		
Theaflavin	Q1	2.72	547	155	1.00			1.00		
	Q2	6.02	548	130	0.79	0.60	1.04	0.91	0.67	1.24
	Q3	10.33	548	95	0.54	0.40	0.72	0.68	0.48	0.95
	Q4	17.15	547	107	0.62	0.47	0.83	0.72	0.51	1.02
*p*-value for trend					0.0004			0.0426		
Theaflavin 3-gallate	Q1	1.81	547	152	1.00			1.00		
	Q2	4.59	548	128	0.80	0.61	1.05	0.93	0.68	1.26
	Q3	6.72	548	111	0.67	0.50	0.89	0.90	0.64	1.25
	Q4	10.54	547	96	0.56	0.42	0.75	0.69	0.48	0.98
*p*-value for trend					<0.0001			0.0395		
Theaflavin 3′-gallate	Q1	6.17	547	145	1.00			1.00		
	Q2	20.06	548	124	0.82	0.62	1.08	1.04	0.76	1.42
	Q3	29.28	548	123	0.82	0.62	1.08	1.12	0.81	1.55
	Q4	42.43	547	95	0.60	0.44	0.80	0.80	0.56	1.14
*p*-value for trend					0.0009			0.3429		
Theaflavin 3,3′ digallate	Q1	5.27	547	142				1.00		
	Q2	24.35	548	118	0.79	0.60	1.05	0.91	0.67	1.25
	Q3	36.33	548	130	0.91	0.69	1.20	1.22	0.89	1.69
	Q4	47.04	547	97	0.63	0.47	0.85	0.84	0.59	1.20
*p*-value for trend					0.0101			0.8335		
Flavanones										
Hesperidin	Q1	0.83	549	138	1.00			1.00		
	Q2	2.56	544	111	0.76	0.57	1.01	0.83	0.61	1.14
	Q3	6.96	546	128	0.92	0.69	1.21	0.87	0.63	1.19
	Q4	12.34	551	110	0.74	0.56	0.99	0.81	0.58	1.12
*p*-value for trend					0.1644			0.3301		
Naringenin	Q1	0.16	547	144	1.00			1.00		
	Q2	0.38	551	137	0.93	0.71	1.22	1.19	0.87	1.62
	Q3	0.70	554	102	0.64	0.48	0.85	0.71	0.51	0.99
	Q4	1.77	538	104	0.67	0.51	0.90	0.74	0.53	1.03
*p*-value for trend					0.0055			0.0201		
Eriodictyol	Q1	0.00	563	145	1.00			1.00		
	Q2	0.02	513	111	0.80	0.60	1.06	0.87	0.63	1.19
	Q3	0.04	577	134	0.88	0.67	1.16	1.01	0.74	1.38
	Q4	0.05	537	97	0.65	0.48	0.87	0.90	0.64	1.28
*p*-value for trend					0.0186			0.8027		
Isoflavones										
Daidzein	Q1	1.78	547	135	1.00			1.00		
	Q2	3.60	548	129	0.94	0.72	1.25	0.95	0.69	1.30
	Q3	5.96	548	116	0.81	0.61	1.08	0.83	0.59	1.15
	Q4	11.19	547	107	0.74	0.56	0.99	0.74	0.51	1.07
*p*-value for trend					0.0282			0.0832		
Genistein	Q1	2.34	547	140	1.00			1.00		
	Q2	4.51	548	125	0.86	0.65	1.14	0.91	0.66	1.25
	Q3	7.41	548	113	0.75	0.57	0.99	0.75	0.54	1.04
	Q4	13.95	547	109	0.72	0.54	0.96	0.73	0.51	1.06
*p*-value for trend					0.0278			0.0885		
Glycitein	Q1	0.69	547	129	1.00			1.00		
	Q2	1.31	548	127	0.98	0.74	1.30	1.04	0.76	1.43
	Q3	2.07	548	125	0.95	0.72	1.26	0.98	0.71	1.36
	Q4	3.79	547	106	0.77	0.58	1.04	0.83	0.57	1.20
*p*-value for trend					0.0626			0.2374		
Coumestrol	Q1	0.02	547	139	1.00			1.00		
	Q2	0.03	548	114	0.77	0.58	1.02	0.77	0.56	1.06
	Q3	0.05	548	126	0.87	0.66	1.15	0.95	0.68	1.31
	Q4	0.11	547	108	0.72	0.54	0.95	0.72	0.50	1.04
*p*-value for trend					0.0766			0.1683		
Formonnetin	Q1	0.00	547	126	1.00			1.00		
	Q2	0.01	548	133	1.05	0.79	1.39	1.18	0.86	1.62
	Q3	0.01	548	110	0.83	0.62	1.11	0.91	0.66	1.28
	Q4	0.03	547	118	0.90	0.68	1.20	1.09	0.77	1.55
*p*-value for trend					0.5079			0.5766		
Biochanin A	Q1	0.00	547	129	1.00			1.00		
	Q2	0.01	548	130	0.99	0.75	1.31	1.03	0.75	1.42
	Q3	0.02	548	110	0.81	0.61	1.08	0.86	0.61	1.20
	Q4	0.03	547	118	0.88	0.66	1.17	1.03	0.72	1.48
*p*-value for trend					0.1913			0.8488		
Anthocyanins										
Cyanidin	Q1	1.32	547	141	1.00			1.00		
	Q2	3.30	548	142	1.02	0.77	1.33	1.00	0.74	1.36
	Q3	6.89	548	104	0.68	0.51	0.91	0.74	0.53	1.03
	Q4	13.05	547	100	0.65	0.49	0.87	0.66	0.47	0.93
*p*-value for trend					0.0004			0.0055		
Delphinidine	Q1	0.01	547	151	1.00			1.00		
	Q2	0.07	546	121	0.75	0.57	0.98	0.77	0.57	1.05
	Q3	0.20	552	97	0.56	0.42	0.75	0.65	0.47	0.91
	Q4	0.54	545	118	0.73	0.55	0.96	0.77	0.56	1.07
*p*-value for trend					0.0991			0.3163		
Pelargonidine	Q1	0.02	547	156	1.00			1.00		
	Q2	0.09	546	124	0.73	0.56	0.96	0.83	0.61	1.13
	Q3	0.16	552	109	0.62	0.47	0.82	0.67	0.49	0.92
	Q4	0.48	545	98	0.55	0.41	0.73	0.64	0.46	0.90
*p*-value for trend					0.0003			0.0199		
Peonidine	Q1	0.10	530	139	1.00			1.00		
	Q2	0.48	513	117	0.83	0.62	1.10	1.02	0.74	1.40
	Q3	0.83	576	118	0.72	0.55	0.95	0.80	0.58	1.10
	Q4	2.50	571	113	0.70	0.52	0.92	0.78	0.56	1.08
*p*-value for trend					0.029			0.115		

Model 1 adjusted for age; model 2 adjusted for age, BMI, education level, household income, marital status, job, current alcohol drinking, current smoking, physical activity, chronic disease status (diabetes, hypertension, cancers, or cardiovascular diseases), sleep duration, family history of depression, stress, menopause status, and total energy intake; OR, odds ratio; CI, confidence interval; Q, quartile.

**Table 5:** Association between total carotenoid and carotenoid subclass intake and prevalence of depressive symptoms in multivariate-adjusted logistic regression analysis.

Variables	Quartile	Median	No. of Total	No. of Cases	Model 1			Model 2		
					OR	95% CI Lower Upper		OR	95% CI Lower Upper	
Total Carotenoids	Q1	11.61	396	151	1.00			1.00		
	Q2	18.03	423	125	0.77	0.58	1.01	0.72	0.53	0.99
	Q3	25.65	444	104	0.61	0.46	0.82	0.67	0.48	0.94
	Q4	38.29	440	107	0.64	0.48	0.84	0.70	0.47	1.04
*p*-value for trend					0.0001			0.0604		
α-carotene	Q1	0.15	412	135	1.00			1.00		
	Q2	0.30	418	130	0.94	0.71	1.24	0.85	0.62	1.17
	Q3	0.48	428	120	0.84	0.63	1.11	0.82	0.59	1.15
	Q4	0.83	445	102	0.69	0.51	0.92	0.74	0.51	1.09
*p*-value for trend					0.0068			0.1635		
β-carotene	Q1	3.27	397	150	1.00			1.00		
	Q2	4.99	429	119	0.74	0.56	0.98	0.82	0.59	1.12
	Q3	6.75	437	111	0.68	0.51	0.90	0.90	0.64	1.27
	Q4	9.56	440	107	0.65	0.49	0.87	0.82	0.55	1.22
*p*-value for trend					0.0041			0.4396		
Lycopene	Q1	0.28	406	141	1.00			1.00		
	Q2	0.82	416	132	0.91	0.69	1.20	0.92	0.68	1.26
	Q3	2.00	434	114	0.76	0.57	1.00	0.80	0.58	1.10
	Q4	4.30	447	100	0.64	0.48	0.86	0.66	0.47	0.92
*p*-value for trend					0.0015			0.0106		
Lutein	Q1	0.71	412	135	1.00			1.00		
	Q2	1.23	427	121	0.86	0.65	1.14	0.95	0.69	1.31
	Q3	1.88	434	114	0.80	0.60	1.06	0.99	0.71	1.39
	Q4	3.42	430	117	0.84	0.63	1.11	1.01	0.70	1.45
*p*-value for trend					0.2813			0.8717		
Zeaxanthin	Q1	0.07	403	144	1.00			1.00		
	Q2	0.13	431	117	0.76	0.58	1.01	0.75	0.55	1.04
	Q3	0.22	423	125	0.83	0.63	1.10	0.86	0.62	1.19
	Q4	0.37	446	101	0.64	0.48	0.86	0.63	0.44	0.90
*p*-value for trend					0.0077			0.028		
β-cryptoxanthin	Q1	0.12	416	131	1.00			1.00		
	Q2	0.21	406	142	1.11	0.85	1.47	1.05	0.77	1.44
	Q3	0.32	434	114	0.84	0.63	1.12	0.76	0.54	1.07
	Q4	0.50	447	100	0.72	0.53	0.96	0.75	0.52	1.09
*p*-value for trend					0.004			0.0542		
Capsaicin	Q1	4.70	401	146	1.00			1.00		
	Q2	8.02	424	124	0.79	0.60	1.05	0.82	0.60	1.13
	Q3	12.90	436	112	0.70	0.53	0.93	0.77	0.55	1.08
	Q4	20.52	442	105	0.65	0.49	0.86	0.70	0.48	1.02
*p*-value for trend					0.0035			0.0782		

Model 1 adjusted for age; model 2 adjusted for age, BMI, education level, household income, marital status, job, current alcohol drinking, current smoking, physical activity, chronic disease status (diabetes, hypertension, cancers, or cardiovascular diseases), sleep duration, family history of depression, stress, menopause status, and total energy intake; OR, odds ratio; CI, confidence interval; Q, quartile.

**Table 6:** Docking score (EFEB) of selected molecules with MAOA and MAOB.

No	Name of Compound	EFEB in MAOA	EFEB in MAOB
1	(−) Epicatechin-3-gallate	−12.73	−13.84
2	Quercetin	−11.43	−10.91
3	Myricetin	−10.83	−10.91
4	Luteolin	−10.81	−10.84
5	Eriodictyol	−10.78	−10.98
6	Kaempferol	−10.37	−9.97
7	Delphinidine	−10.27	−10.4
8	Petunidine	−10.25	−10.38
9	Capsaicin	−10.24	−10.43
10	Biochanin A	−10.12	−10.37
11	Cyanidin	−9.98	−9.86
12	Naringenin	−9.89	−10.05
13	Apigenin	−9.84	−9.94
14	Peonidine	−9.69	−10.41
15	Glycitein	−9.68	−9.59
16	Formonnetin	−9.67	−10.26
17	Genistein	−9.65	−10.28
18	(+)-Catechin	−9.62	−10.33
19	(+)-Gallocatechin	−9.54	−10.37
20	Coumestrol	−9.47	−10.3
21	Epigallocatechin	−8.96	−13.34
22	Pelargonidine	−8.96	−9.06
23	Daidzein	−8.96	−9.72
24	Theaflavin	59.64	−7.74

EFEB: estimated free energy of binding, MAOA: monoamine oxidase A, MAOB: monoamine oxidase B.

**Table 7:** Docking score (EBEF) of the selected carotenoids with BDNF.

Sr. No	Name of Compound	EFEB in BDNF
1	α-Carotene	−7.24
2	β-Cryptoxanthin	−6.79
3	Lycopene	−6.47
4	β-Carotene	−6.18
5	Capsaicin	−6.17
6	Zeaxanthin	−6.10
7	Lutein	−6.06

EFEB: estimated free energy of binding, BDNF: brain-derived neurotrophic factor.

## Data Availability

All data are reported in this manuscript.
